# Supplementary material for: The left posterior angular gyrus is engaged by autobiographical recall not object-semantics, or event-semantics: Evidence from contrastive propositional speech production
Source: Imaging Neurosci (Camb). 2024 Mar 25;2:imag-2-00116. doi: 10.1162/imag_a_00116 (PMC12247623; doi:10.1162/imag_a_00116)
Supplement: Supplementary Material [file imag_a_00116-supp.pdf]

Supplementary

Table S1. A description of the studies included in the metaanalysis.

*An “unconstrained” task is that in which the participants were free to respond in any way they choose, in contrast to a “constrained” tasks whereby participants were restricted in how they responded (e.g. the response must avoid certain words).

Study	Task	Contrast	Technique	N. Participants	Activated the AG
Braune et al. 2001	Generate a personal story	Speech > oral-motor baseline	PET	12	Yes
Blank, Scott, Murphy, Warburton, and Wise (2002)	Generate a personal story	Speech > counting + nursery rhyme recitation	PET	8	Yes
Haller, Radue, Erb, Grodd, and Kircher (2005)	Generate a sentence based on three words	Sentence generation > word reading	fMRI	15	No
		Sentence generation > sentence reading			No
Kemeny, Ye, Birn, and Braun (2005)	Generate a sentence based on verb	Sentence generation > syllable repetition (pa-ta-ka)	fMRI	6	No
Troiani et al. (2008)	Generate a story based on pictures	Sentence generation > story viewing + syllable repetition (pa-da-ka)	fMRI	15	No
Tremblay and Small (2011)	Object description	Sentence generation > picture viewing	fMRI	21	No
Menenti et al. (2011)	Picture description	Repeated words in production > repeated words in comprehension	fMRI	20	No
Geranmayeh et al. (2012)	Unconstrained	Define nouns > tongue movement	fMRI	19	No
Grande et al. (2012)	Constrained (avoid frequent words)	Sentence generation > rest	fMRI	18	No
Menenti, Segaert, and Hagoort (2012)	Picture description	Novel semantics > repeated semantics	fMRI	20	No
		Novel words > repeated words			
		Novel syntax > repeated syntax			
Menenti, Petersson, and Hagoort (2012)	Picture description	Novel words > repeated words	fMRI	24	No
		Novel meaning > repeated meaning			
Geranmayeh, Wise, Mehta, and Leech (2014)	Define a noun	Define a noun > counting	fMRI	24	No
Schönberger et al. (2014)	Generate a sentence	Generation of simple complete sentences > sentences missing verb	fMRI	15	Yes
Simmonds, Leech, Collins, Redjep, and Wise (2014)	Unconstrained	Overt noun definition > rest (look at a row of Xs)	fMRI	17	No
Matchin and Hickok (2016)	Active or passive	Sentence generation > word lists	fMRI	20	No

Dhanjal, Handunnetthi, Patel, and Wise (2008)	Define a noun	Define a noun > counting	fMRI	21	No
Brownsett and Wise (2010)	Self-referential narrative	Self-referential speech > syllables	PET	13	Yes
Humphreys and Gennari (2014)	Sentence completion	Sentence completion > reading	fMRI	17	No
Braun et al. (1997)	Self-referential speech	Production tasks > mouth movements	PET	8	Yes
Kircher et al. (2004)	Picture description	Free speech > rest	fMRI	6	No
Haller, Radue, Erb, Grodd, and Kircher 2005	Sentence generation based on key words	Sentence generation > word reading	fMRI	15	No
Tremblay and Small (2011)	Generate sentence from pictures	Sentence generation > rest	fMRI	21	No
Kemeny, Ye, Birn, and Braun (2005)	Sentence generation based on verb	Sentence generation > rest	fMRI	6	No
AbdulSabur, Xu, Chow, Baxter, Carson, and Braun (2014)	Retell a previously presented story	Narrative production > nursery rhyme	fMRI/PET	18	No
Yuan, Major-Girardin, and Brown (2018)	Free narrative production	Narrative production > picture description	fMRI	24	Yes

Table S2. A complete list of experimental stimuli in include in the fMRI task.

A list of experimental stimuli		
Condition	ItemN	Question
autobio	1	Describe the last time you arranged a holiday
autobio	2	Describe the last time you used a bike
autobio	3	Describe your most recent journey to work/university
autobio	4	Describe the last time you bought a present
autobio	5	Describe the last time you caught a train
autobio	6	Describe the last time you sent an email
autobio	7	Describe the last time you brushed your teeth
autobio	8	Describe the last time you bought clothes or shoes
autobio	9	Describe the last time you made the bed
autobio	10	Describe the last time you took a photograph
autobio	11	Describe the last time you cleaned the dishes
autobio	12	Describe the last time you went to a shopping centre
autobio	13	Describe the last time you went to a restaurant
autobio	14	Describe the last time you went to the pub
autobio	15	Describe the last time you met with a friend
autobio	16	Describe the last time you sent a message using your phone
autobio	17	Describe the last time you had lunch
autobio	18	Describe the last time you caught a bus
autobio	19	Describe what you did for dinner last night
autobio	20	Describe the last time you went to a supermarket or food shop
autobio	21	Describe your most recent trip to a café
autobio	22	Describe the last time you went to a party
autobio	23	Describe the last time you used a kettle
autobio	24	Describe the last time you ate desert
autobio	25	Describe the last time you spoke on the phone
autobio	26	Describe the last time you went to the airport
autobio	27	Describe the last time you watched a TV show or movie
autobio	28	Describe the last time you had breakfast
autobio	29	Describe the last time you had coffee or tea
autobio	30	Describe the last time you had your haircut
autobio	31	Describe the last time you wrote an essay or paper
autobio	32	Describe the last time you cleaned the kitchen
autobio	33	Describe the last time you did the laundry
autobio	34	Describe the last time you used the oven

autobio	35	Describe the last time you ate fruit or vegetables
autobio	36	Describe the last time you travelled by car
semEvent	1	Describe what you would typically do when visiting a restaurant
semEvent	2	Describe what you would typically do when visiting a pub
semEvent	3	Describe what you would typically do to arrange to meet with a friend
semEvent	4	Describe how you would typically write a text message
semEvent	5	Describe what you would typically do to make a jam sandwich
semEvent	6	Describe what you would typically do to catch a bus
semEvent	7	Describe what you would typically do to cook pasta
semEvent	8	Describe what you would typically do when visiting the supermarket
semEvent	9	Describe what you would typically do in a café
semEvent	10	Describe what you would typically do at a party
semEvent	11	Describe how you would typically use a kettle
semEvent	12	Describe how you would typically make a cake
semEvent	13	Describe how you would typically make a phone call
semEvent	14	Describe what typically happens when you visit an airport
semEvent	15	Describe what you would typically do when visiting the cinema
semEvent	16	Describe what you would typically do to fry an egg
semEvent	17	Describe how you would typically make a cup of tea
semEvent	18	Describe what typically happens when you go for a haircut
semEvent	19	Describe how you would typically write an essay
semEvent	20	Describe what you would typically do to clean the kitchen
semEvent	21	Describe how you would typically do the laundry
semEvent	22	Describe how you would typically use an oven
semEvent	23	Describe how you would typically chop vegetables
semEvent	24	Describe what you typically do to start driving a car
semEvent	25	Describe what you would typically do in order to arrange a holiday
semEvent	26	Describe how you would typically ride a bike
semEvent	27	Describe how you typically travel to work
semEvent	28	Describe what you would typically do in order to buy a present
semEvent	29	Describe what you would typically do when catching the train
semEvent	30	Describe what you would typically do to send an

		email
semEvent	31	Describe what you would typically do to brush your teeth
semEvent	32	Describe what you would typically do when clothes shopping
semEvent	33	Describe how you would typically make the bed
semEvent	34	Describe what you would typically do to take a photograph
semEvent	35	Describe how you typically cleans the dishes
semEvent	36	Describe what you would typically do at a shopping centre
semObj	1	What is a phone?
semObj	2	What is an airport?
semObj	3	What is a movie?
semObj	4	What is breakfast?
semObj	5	What is coffee?
semObj	6	What is a haircut?
semObj	7	What is an essay?
semObj	8	What is a kitchen?
semObj	9	What is laundry?
semObj	10	What is an oven?
semObj	11	What is fruit?
semObj	12	What is a car?
semObj	13	What is a holiday?
semObj	14	What is a bike?
semObj	15	What is a University?
semObj	16	What is a present?
semObj	17	What is a train?
semObj	18	What is an email?
semObj	19	What is a toothbrush
semObj	20	What are clothes?
semObj	21	What is a bed?
semObj	22	What is a photograph?
semObj	23	What is a dish?
semObj	24	What is a shopping centre?
semObj	25	What is a restaurant?
semObj	26	What is a pub?
semObj	27	What is a friend?
semObj	28	What is a text message?
semObj	29	What is lunch?
semObj	30	What is a bus?

semObj	31	What is dinner?
semObj	32	What is a supermarket?
semObj	33	What is a café?
semObj	34	What is a party?
semObj	35	What is a kettle?
semObj	36	What is desert?

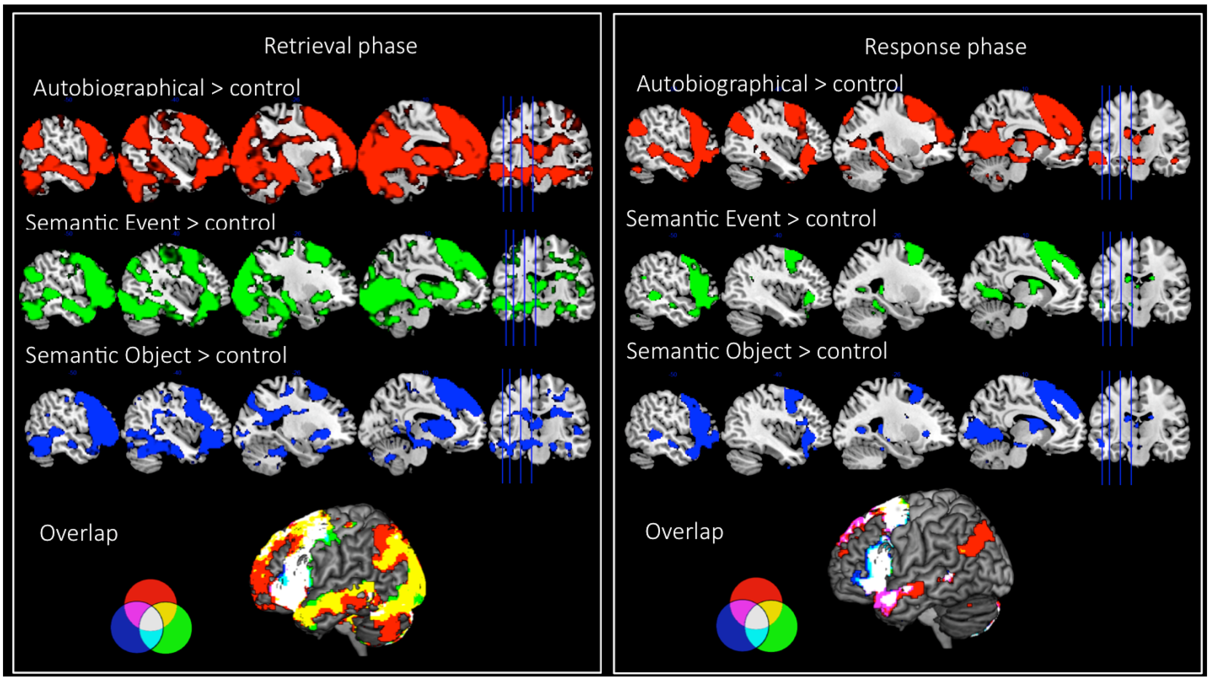

Figure S3. The fMRI results from each experimental task > control task from the "retrieval phase" (left), "response phase" (right) (yellow, white, cyan, violet reflect overlapping activations). Voxel height threshold $p < .001$, cluster corrected using FWE $p < .05$

Table S4. The peak coordinates from significant clusters in the metaanalysis of 25 speech production studies.

Region		Coordinates		
Parietal cluster	SPL	-21	-67	49
	Posterior cingulate/precuneus	2	-57	12
	IPS	-30	-53	44
	Precuneus	-7	-59	29
	Precuneus	-7	-59	50
	AG	-52	-59	26
	AG	-44	-74	30
Temporal clusters	MTG	-59	-46	4
	SMA	-4	12	59
Frontal clusters	Precentral gyrus	-44	3	50
	IFG BA44	-47	20	8

Figure S5. The locus of activation for the contrast of Autobiographical > rest (red) and the Semantic event > rest (green), overlap (yellow) during the "retrieval phase" (as shown in Figure 2) relative to the anatomical boundaries of PGa (anterior) and PGp (posterior), as defined by Caspers et al. (2008).

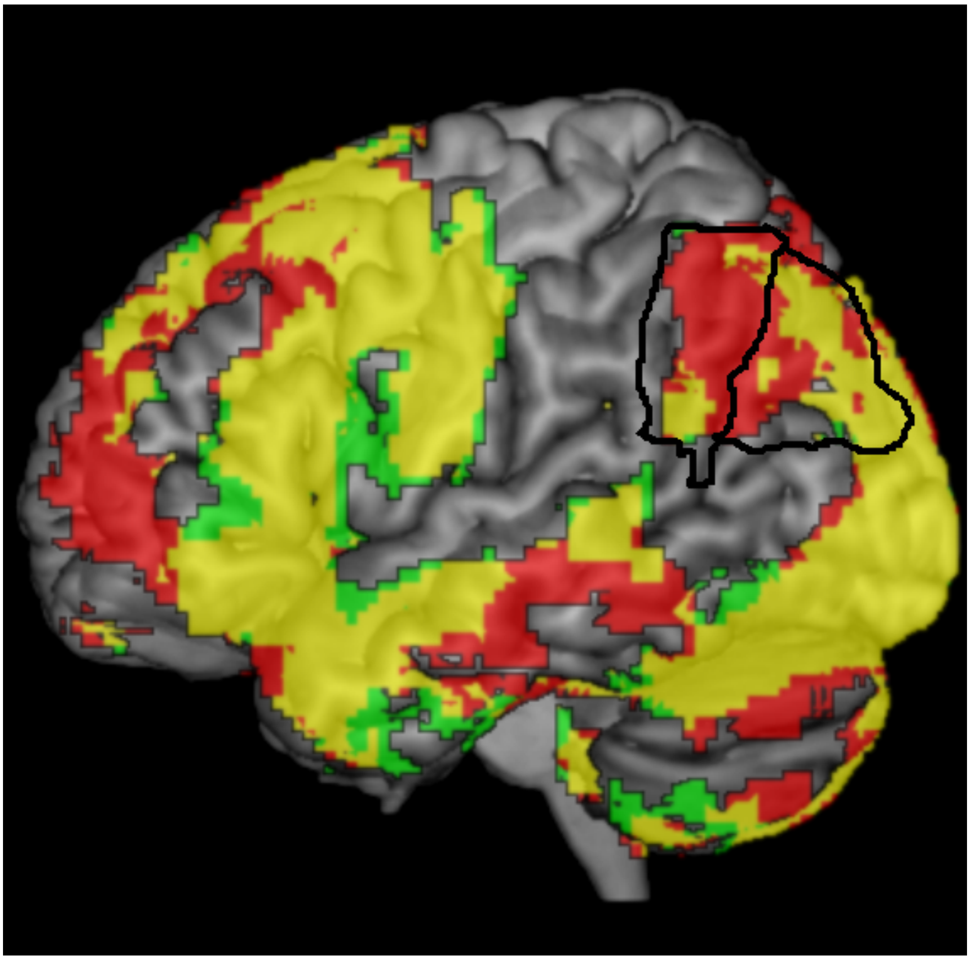

Table S6. The fMRI results from each task > rest and the overlap in activation from the “retrieval phase”. Voxel height threshold p < .001, cluster corrected using FWE p < .05

Contrast	Cluster size	T	MNI coordinates
autobiographical > rest	15988	18.83	-16 -60 10
		17.38	4 -72 -28
		16.01	14 -84 -4
	1342	12.51	-16 16 54
		12.4	-10 22 56
		11.7	-10 44 36
	267	12.45	-30 34 -2
		9.77	-38 36 -10
		9.18	-36 46 0
	347	11.76	-34 -68 38
		10.03	-46 -70 32
		9.45	-30 -78 42
	312	11.64	-52 -2 24
		10.36	-46 -8 26
		10.12	-52 -10 36
	206	11.38	-38 -50 20
		11.27	-44 -50 26
		9.52	-36 -48 28
	691	11.08	16 20 30
		10.24	22 8 38
		10.21	28 0 38
	121	11.05	32 44 -2
		10.81	36 38 -6
	302	10.98	36 -30 -10
		10.86	38 -18 -18
		10.27	32 -12 -18
	189	10.4	-50 18 18

		9.16	-42	16	40
		9.04	-40	14	24
	144	9.94	-14	-38	30
		9.72	-4	-40	34
		9.7	-16	-44	36
object semantics > rest	40374	14.13	-12	-62	-22
		13.7	34	36	-4
		13.56	-6	10	58
	104	8.66	20	-12	-4
		8	18	-6	2
	125	8.08	-10	44	34
		7.79	-8	52	32
		6.87	-8	42	46
	111	7.98	-56	-38	4
		6.83	-64	-40	6
event semantics > rest	45385	15.73	6	-74	-26
		15.19	34	36	-4
		14.33	14	-86	-4
	147	8.37	-52	-38	4
		6.64	-66	-38	2
	33	7.93	-22	-20	42
		6.43	-18	-20	50
	116	7.85	40	18	-26
		6.93	34	14	-32
		6.92	32	12	-40
Autobiographical > event semantics	11415	11.2	0	-64	28
		10.47	-10	-58	30
		10.13	4	-56	28
	11875	10.02	-4	58	-6
		9.09	-6	40	-4
		8.53	-4	54	16

	1332	6.71	-46 -60 30
		6.22	-44 -74 42
		5.37	-52 -70 30
	991	6.33	-58 -14 -14
		5.66	-58 -6 -18
		5.47	-68 -16 -14
	1529	6.28	60 -8 -16
		5.98	52 16 -32
		5.86	30 20 -16
	1344	6.08	50 -60 26
		5.59	44 -72 40
		5.25	52 -72 22
	550	5.78	-4 -52 -46
		5.76	10 -48 -44
		4.72	0 -58 -50
	261	5.37	-30 10 -16
		4.28	-38 14 -26
		3.26	-40 14 -12
	580	4.63	-28 -84 -36
		4.45	-40 -78 -38
		4.19	-8 -84 -38
	274	4.55	26 -86 -28
		3.99	6 -86 -20
		3.96	38 -86 -30
Semantic event > autobiogrphical	1266	5.53	-48 -14 38
		5.46	-52 -8 28
		5.09	-58 -8 18
	970	5.12	62 -6 24
		5.07	52 -2 24
		4.94	52 -8 30
	178	4.39	-48 34 2

	265	4.13	-50 -62 -12
		3.57	-42 -54 -6
		3.45	-42 -50 -14
Semantic event > semantic Object	4240	8.32	8 -60 54
		8.27	-8 -68 50
		8.24	-10 -62 58
	2739	8.15	26 14 58
		6.52	32 6 66
		5.47	36 26 52
	3024	7.59	56 -40 46
		6.87	56 -50 20
		6.67	60 -48 32
	1328	6.83	-60 -38 44
		4.07	-48 -46 60
		3.97	-34 -42 42
	801	6.78	-24 4 60
		4.77	-24 -8 64
		3.68	-20 8 72
	235	6.23	48 -40 -38
		4.81	34 -42 -44
	460	5.94	-42 -42 -42
		5.77	-34 -44 -40
		4.16	-40 -58 -42
	189	5.9	-32 -40 -12
	1073	5.76	-58 -60 10
		5.22	-22 -62 18
		5.12	-48 -64 4
	263	5.73	-34 -86 32
		4.96	-38 -88 24
	387	5.57	60 -30 -18
		5.2	58 -20 -14

		4.16	50	-30	-14
	191	5.25	20	-58	16
	275	4.99	-54	-10	26
		4.82	-46	-12	22
		3.42	-64	-10	18
	200	4.83	-40	-10	-4
		4.61	-42	0	-2
		3.55	-48	-12	2
	209	4.53	30	54	-14
		4.11	22	54	-16
		3.79	22	36	-22
	173	3.99	18	58	22
		3.7	30	56	14
Semantic object > semantic event	414	7.68	-16	-86	-12
		7.56	-10	-90	-2
	607	6.97	-28	-88	6
		6.77	-22	-94	14
		6.77	-24	-88	20
	578	6.88	18	-96	-2
		6.67	12	-86	-2
		6.29	36	-86	0
	128	6.67	28	-82	20

S7. ROI analyses from the response phase of the experiment: the AG showed significantly greater activation relative to all other conditions (all ts > 6.29, all ps < .000). The AG did not differ in response to the semantic-events and semantic-objects (t(27) = 1.19, p =.25), although both conditions differed to control (t(27) = 3.40, p < .005). With the exception of the autobiographical condition, all conditions showed significant deactivation relative to rest (all ts > -2.90, all ps < .005). The same pattern was found for the full DMN. Unlike the AG, the ATL showed significant great activation for all tasks relative to control (all ts > 6.22, all ps < .001), but no significant difference between conditions (all ts < 1.27, all ps > .22).
